# Cost-effectiveness analysis of tislelizumab plus chemotherapy as the first-line treatment for advanced or metastatic esophageal squamous cell carcinoma in China

**DOI:** 10.3389/fphar.2024.1225076

**Published:** 2024-05-15

**Authors:** Yanhong Liu, Rong Shao

**Affiliations:** School of International Pharmaceutical Business, China Pharmaceutical University, Nanjing, China

**Keywords:** cost-effectiveness analysis, tislelizumab, advanced OSCC, Chinese healthcare system, partitioned survival model

## Abstract

**Introduction:**

First-line treatment with tislelizumab plus chemotherapy has shown clinical benefits for patients with advanced or metastatic esophageal squamous cell carcinoma (OSCC) in China, while its economic burden is unknown. This study aimed to evaluate the cost-effectiveness of tislelizumab plus chemotherapy from the perspective of the Chinese healthcare system.

**Methods:**

We constructed a partitioned survival model to compare the cost-effectiveness of tislelizumab plus chemotherapy with chemotherapy in patients with advanced OSCC. Patient characteristics and clinical outcomes were extracted from RATIONALE-306. Costs, quality-adjusted life-years (QALYs), and incremental cost-effectiveness ratios (ICERs) were selected as the study outcomes. Sensitivity analysis and subgroup analysis were conducted to test the stability of the results.

**Results:**

Tislelizumab plus chemotherapy provided additional 0.48 QALYs with the incremental cost of $16,587.2 than chemotherapy, of which ICER was $34,699.72 per QALY. When the willingness-to-pay threshold was set as $37,260, the novel therapy had a probability of 77% to be cost-effective. Our base-case analysis results were sensitive to utilities of progression-free survival and progression of disease. Our subgroup analysis showed that the novel therapy was associated with cost-effectiveness in patients with a high expression of PD-L1.

**Conclusion:**

Tislelizumab plus chemotherapy was likely to be more cost-effective compared with chemotherapy in the first-line therapy of advanced OSCC from the perspective of the Chinese healthcare system. Our findings can provide clinicians and decision-makers with evidence of the cost-effectiveness of tislelizumab.

## Introduction

Esophageal cancer ranked as the seventh highest in incidence and sixth highest in mortality among all cancers worldwide in 2020 (Sung et al., 2021). Esophageal squamous cell carcinoma (OSCC) and esophageal adenocarcinoma represent the predominant subtype of esophageal cancer, with the former accounting for approximately 85% of the cases (Arnold et al., 2017). More than half of the number of patients with OSCC is from China (Arnold et al., 2020; The Lancet, 2020). Every year, nearly 320,000 new cases are diagnosed in China (Cao et al., 2021). For decades, standard fluoropyrimidine or paclitaxel plus cisplatin-based chemotherapy has been recommended as first-line treatment for patients with advanced or metastatic OSCC. Meanwhile, only a median overall survival (OS) of 7.0–13.0 months could be obtained based on data from several clinical studies (Ilson et al., 1998; Petrasch et al., 1998; Zhang et al., 2008; Ajani et al., 2019; Shah et al., 2023). Therefore, discovering revolutionary first-line treatment strategies to improve clinical therapy in these populations are needed.

Recently, with the emergence of immune checkpoint inhibitors (ICIs) like programmed death 1 (PD-1) or programmed death-ligand 1 (PD-L1), the landscape of cancer treatment has been significantly altered (Shao et al., 2022). Tislelizumab is a humanized IgG4 monoclonal anti-body with high affinity and binding specificity for PD-1, which has shown clinical efficacy in many cancers (Xu J. et al., 2023). As of May 2023, tislelizumab has been approved by the National Medical Products Administration (NMPA) of China for the treatment of nine indications (e.g., unresectable hepatocellular carcinoma and non-small-cell lung cancer) (Qin et al., 2019; Lu et al., 2021; Wang et al., 2021; Zhou et al., 2023). Note that tislelizumab has also been approved by NMPA for second-line treatment of OSCC.

Recently, the global, randomized, placebo-controlled, phase III trial RATIONALE-306 reported the efficacy and safety of tislelizumab *versus* a placebo in combination with chemotherapy (platinum agent plus fluoropyrimidine, capecitabine, or paclitaxel) as the first-line treatment of advanced or metastatic OSCC (Xu J. et al., 2023). The results revealed that tislelizumab plus chemotherapy markedly prolonged the median progression-free survival (PFS) (7.3 months vs. 5.6 months, HR = 0.62, 95% confidence interval 0.52–0.75) and OS (17.2 months vs. 10.6 months; HR = 0.66, 95% confidence interval 0.54–0.80) when compared with placebo plus chemotherapy. Therefore, tislelizumab plus chemotherapy has the potential to be an alternative for treating advanced OSCC. The price of tislelizumab in the National Reimbursement Drug List (NRDL) is $199.64/100 mg (Cancer361, 2023). However, tislelizumab has not been approved by NMPA for first-line treatment of OSCC yet.

Although the clinical results can be encouraging, physicians and decision-makers should not ignore the evidence of cost-effectiveness since combination therapies have a relatively higher cost when compared with chemotherapy alone. Cost-effectiveness analysis serves as a decision-making framework designed to compare alternative projects or programs that share the same objective(s). The comparison is made on the basis of the monetary costs required to achieve a specific level of output or outcome, which is already deemed beneficial (Palkovics, 1989). As a result, the purpose of this study was to investigate the cost-effectiveness of tislelizumab plus chemotherapy as the first-line treatment for advanced or metastatic OSCC from the perspective of the Chinese healthcare system.

## Materials and methods

### Model structure

**FIGURE 1:**
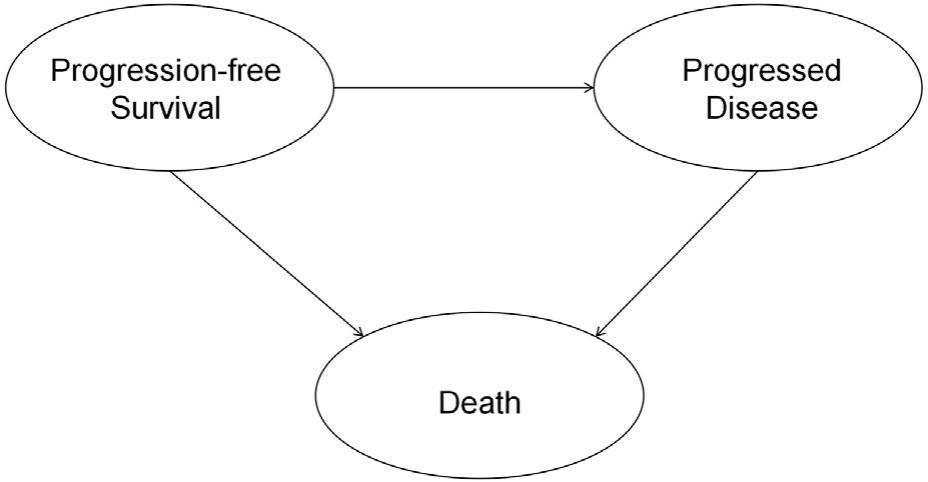
Schematic diagram of the partition survival model structure.

This study was reported following the Consolidated Health Economic Evaluation Reporting Standards (CHEERS) reporting guideline (Husereau et al., 2022). This economic evaluation was conducted from the perspective of the Chinese healthcare system. The results can be generalized to the situation in China since 75% of the patients in RATIONALE-306 were Asians. A partitioned survival model with three states was constructed for this cost-effectiveness analysis (Williams et al., 2017). The model included three health states: progression-free survival (PFS), progressed disease (PD), and death (D) (Figure 1). Patients in the model move over time from one health state to another, with the probabilities of these transitions being determined by survival functions. These survival functions are partitioned. The time horizon was set as 10 years so that 99% of the patients died in both treatment arms, and the cycle length was set as 21 days (Shao et al., 2022). The primary outcomes were cost, quality-adjusted life-years (QALYs), and the incremental cost-effectiveness ratio (ICER). Both costs and QALYs were discounted at an annual rate of 5% (Guoen, 2020). The willingness-to-pay (WTP) threshold was set as $37,260 (three times the Chinese GDP *per capita* in 2022) as per the QALY gained (National Bureau of Statistics, 2021). The statistical analysis was conducted through R 4.1.2 (https://www.r-project.org/), and the economic evaluation model was constructed using Microsoft Excel 2019 (Redmond, Washington, United States).

### Patients and intervention

We included patients who were ≥18 years of age with histologically confirmed advanced or metastatic OSCC. Other key characteristics were the same as in the RATIONALE-306 trial (Xu J. et al., 2023). The included patients received tislelizumab or placebo intravenously at a dose of 200 mg every 3 weeks (one cycle). Chemotherapy included cisplatin (75 mg/m^2^ every cycle) or oxaliplatin (130 mg/m^2^ every cycle) plus paclitaxel (175 mg/m^2^ every cycle) or 5-fluorouracil (750–800 mg/m^2^ continuous administration for 5 days in each cycle) or capecitabine (1,000 mg/m^2^ orally twice daily on days 1–14) (Xu J. et al., 2023). Note that oxaliplatin substitution was not permitted in the treatment guidelines in China (Xu J. et al., 2023). Therefore, only cisplatin was considered in this study. According to the chemotherapy exposure in the patients in the RATIONALE-306 trial, we considered a maximum of six cycles of chemotherapy (Xu J. et al., 2023). If the disease progressed or intolerable toxicity occurred, the patients could alter from the first-line treatments to the second-line treatments. In RATIONALE-306, 48% (157/326) patients in the tislelizumab plus chemotherapy group and 55% (177/323) in the placebo plus chemotherapy group received subsequent systemic anticancer therapies; subsequent immunotherapy was provided to 46 (14%) and 72 (22%) patients, respectively. However, detailed information on the subsequent therapies was not reported (Xu J. et al., 2023). Therefore, according to the recent Chinese guideline from the Chinese Society of Clinical Oncology, we assumed the subsequent therapies as the best supportive care (BSC), camrelizumab (immunotherapy), and docetaxel (chemotherapy) (Wang and Han, 2020).

### Clinical data

We extracted the PFS and OS data through GetData Graph Digitizer (http://getdata-graph-digitizer.com) from the Kaplan–Meier (KM) curves of the RATIONALE-306 trial because we did not have the individual patient data (IPD). Then, the method of Guyot et al. was used to reconstruct the IPD (Guyot et al., 2012). We validate our reconstructed models by comparing them with the original KM curves, and the results showed that the generated PFS and OS curves closely resemble those provided in the RATIONALE-306 trial. We considered the following parametric survival models: exponential, Weibull, Gompertz, gamma, log-logistic, log-normal, and generalized gamma models (Shao et al., 2023). The minimum of the Akaike information criterion (AIC) goodness-of-fit statistics and visual inspection were used to determine the best fit of the PFS and OS curves (Jrgensen, 2004; Djalalov et al., 2019). The goodness-of-fit results are shown in Supplementary Tables S1 and S2. The validation plot and survival distribution are also shown in Supplementary Figures S1–S4. The detailed clinical information is given in Table 1.

**TABLE 1:** Parameters input.

Parameter	Value	Range	Source
Cost			
Cost of tislelizumab	199.64	(159.71,199.64)	MENET, 2022; Yaoch (2022)
Cost of cisplatin per unit (10 mg)	1.35	(1.38,1.47)	
Cost of cisplatin per unit (30 mg)	2.77	(3.01,4.4)	
Cost of paclitaxel per unit (100 mg)	25.8	(27,28)	
Cost of 5-fluorouracil	8.41	(6.72,10.09)	
Cost of camrelizumab	373.43	(298.74,448.11)	
Cost of docetaxel	23.48	(22.67,23.76)	
Cost of best supportive care	116.14	(92.91, 139.36)	Zhang et al. (2021)
Routine follow-up cost per cycle	51.07	(40.86, 61.29)	
Cost of laboratory tests and radiological examinations	247.56	(198.05, 297.07)	
Cost of salvage therapy per cycle	443.21	(354.57, 531.85)	
Cost_end of life care	1460.30	(1168.24, 1752.36)	
Cost_decreased neutrophil count	107.28	(51.11,357.8)	Shao et al. (2022), Zhou et al. (2022), Liu et al. (2023)
Cost_decreased white blood cell count	107.28	(51.11,357.8)	
Cost_anemia	129.43	(106.73,160.1)	
Cost_neutropenia	115.01	(92.01,138.01)	
Cost_hypokalemia	107	(80,134)	
Cost_hyponatremia	107	(80,134)	
Utility			
Utility_PFS	0.75	(0.6, 0.9)	Marguet et al. (2021)
Utility_PD	0.67	(0.54, 0.8)	
Disutility_decreased neutrophil count	0.2	(0.16,0.24)	Shao et al. (2022), Zhou et al. (2022), Liu et al. (2023)
Disutility_decreased white blood cell count	0.2	(0.16,0.24)	
Disutility_anemia	0.07	(0.06,0.09)	
Disutility_neutropenia	0.09	(0.07,0.11)	
Disutility_hypokalemia	0.08	(0.06,0.1)	
Disutility_hyponatremia	0.08	(0.06,0.1)	
Risk of AEs (≥ grade 3)			
Tislelizumab group			
Decreased neutrophil count	0.31	(0.24,0.37)	Xu et al. (2023a)
Decreased white blood cell count	0.11	(0.09,0.13)	
Anemia	0.14	(0.11,0.17)	
Neutropenia	0.07	(0.06,0.09)	
Hypokalemia	0.06	(0.04,0.07)	
Hyponatremia	0.07	(0.05,0.08)	
Chemotherapy group			
Decreased neutrophil count	0.06	(0.04,0.07)	Xu et al. (2023a)
Decreased white blood cell count	0.07	(0.05,0.08)	
Anemia	0.33	(0.26,0.39)	
Neutropenia	0.16	(0.12,0.19)	
Other parameters			
Discount rate	0.05	(0, 0.08)	Guoen (2020)
Proportion of paclitaxel used in the tislelizumab group	0.55	(0.44,1)	Xu et al. (2023a)
Proportion of paclitaxel used in the chemotherapy group	0.55	(0.44,1)	
Proportion of subsequent treatment			
Tislelizumab group			
Proportion of subsequent treatment_immunotherapy	0.14	(0.11,0.17)	Xu et al. (2023a)
Proportion of subsequent treatment_chemotherapy	0.48	(0.38,0.58)	
Proportion of subsequent treatment_BSC	0.38	(0.5,0.26)	
Chemotherapy group			
Proportion of subsequent treatment_immunotherapy	0.22	(0.18,0.26)	Xu et al. (2023a)
Proportion of subsequent treatment_chemotherapy	0.55	(0.44,0.66)	
Proportion of subsequent treatment_BSC	0.23	(0.38,0.08)	

Abbreviations: PFS, progression-free survival; PD, progressed disease; AEs, adverse events; BSC, best-supportive care.

### Cost and utility

We only considered direct medical costs in this study, which included costs of acquiring drugs, costs of routine follow-up, costs for the management of adverse events (AEs), and costs for end-of-life care (EOL). Drug prices in 2022 were obtained from two Chinese public databases health data platforms (MENET, 2022; Yaoch, 2022). Follow-up costs and EOL costs were obtained from one published article (Zhang et al., 2021). The prices in RMB were exchanged for US$ with the exchange rate of 6.90 (6 May 2022). Only severe AEs (≥grade 3) with rates over 5% were considered in this study, which included decreased neutrophil count, decreased white blood cell count, anemia, neutropenia, hypokalemia, and hyponatremia. The utilities of PFS and PD states associated with advanced OSCC were 0.75 and 0.67, respectively, which were derived from a published economic evaluation (Marguet et al., 2021). The disutility values according to AEs were included in this analysis. These disutility values were extracted from other published studies (Shao et al., 2022; Zhou et al., 2022). We assumed that all AEs were incurred during the first cycle (Shao et al., 2022). Then, we subtracted the duration-adjusted disutility from the baseline PFS utility. All cost-related and utility-related parameters are shown in Table 1.

### Sensitivity and subgroup analyses

We conducted deterministic sensitivity analysis (DSA) and probabilistic sensitivity analysis (PSA) to test the robustness of the economic evaluation model. In DSA, all parameters were adjusted within the reported 95% confidence intervals (CIs). For parameters not reporting confidence intervals, we assumed a fluctuation of ±20% based on the base-case values. In PSA, a gamma distribution was selected for cost and a beta distribution was selected for probability, proportion, and utility. Scatter plots describing ICER and the cost-effectiveness acceptable curve (CEAC) were obtained by running 10,000 Monte Carlo simulations. In the subgroup analysis, we analyzed the results for patients with the tumor area positivity (TAP) score of ≥10% and <10% for the expression of PD-L1 through the methods of base-case analysis since the RATIONALE-306 trial reported the independent KM curves for OS. Since KM curves for PFS were not reported, we assumed the same PFS as the base-case analysis.

## Results

### Base-case analysis results

Base-case analysis results including cost, QALYs, and ICERs are shown in Table 2. Tislelizumab plus chemotherapy gained 1.38 QALYs at a cost of $34,965.45, while placebo plus chemotherapy yielded 0.9 QALYs and cost $18,378.25. Compared with the placebo, the tislelizumab combination therapy gained incremental QALYs of 0.48 with an incremental cost of $16,587.2. Thus, the ICER between the placebo plus chemotherapy group and the tislelizumab plus chemotherapy group was $34,699.72 per QALY gained, which was slightly lower than the given WTP of $37,260 per QALY gained.

**TABLE 2:** Results of base-case analysis and subgroup analysis.

Drug	Total cost	Total utility	Increment cost	Increment utility	ICER
Base-case analysis					
Placebo plus chemotherapy	18378.25	0.9	—	—	—
Tislelizumab plus chemotherapy	34965.45	1.38	16587.2	0.48	34699.72
Subgroup analysis of TAP score ≥10%					
Placebo plus chemotherapy	18,597.24	0.91	—	—	—
Tislelizumab plus chemotherapy	35504.72	1.4	16907.48	0.49	34553.83
Subgroup analysis of TAP score <10%					
Placebo plus chemotherapy	18151.19	0.88	—	—	—
Tislelizumab plus chemotherapy	30998.97	1.19	12847.78	0.31	41848.57

Abbreviations: TAP, tumor area positivity; ICER, incremental cost-effectiveness ratio.

### Subgroup analysis

Subgroup analysis results including cost, QALYs, and ICERs are shown in Table 2. In the subgroup analysis of patients with a TAP score ≥10%, the tislelizumab combination therapy gained an additional 0.49 QALYs at a cost of additional $16907.48 when compared with placebo plus chemotherapy. The ICER was $34,553.83 per QALY gained, which was similar to the base-case analysis results. However, in the subgroup analysis of patients with a TAP score <10%, the tislelizumab combination therapy yielded an incremental QALY of 0.31 with an incremental cost of $12,847.78. The ICER remained $41,848.57 per QALY gained, which meant that the novel therapy was not cost-effective anymore.

### Sensitivity analysis

**FIGURE 2:**
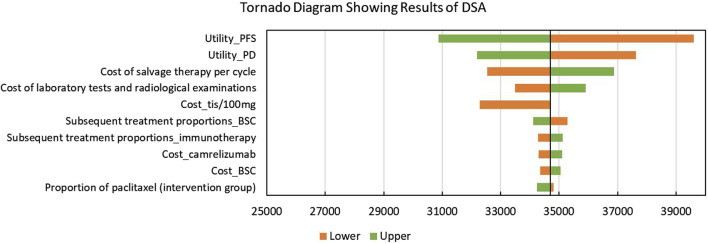
Tornado diagram showing the results of the deterministic sensitivity analysis. Abbreviations: DSA, deterministic sensitivity analysis; PFS, progression-free survival; PD: progressed disease; tis: tislelizumab; BSC, best-supportive care; QALYs, quality-adjusted life years.

The tornado diagram showing the results of DSA is shown in Figure 2. Utilities of PFS and PD were significantly associated with the model outcomes, which even led to the modeled ICERs, exceeding the given WTP threshold. In addition, base-case analysis results were also sensitive to the cost of salvage therapy, laboratory tests and radiological examinations, and tislelizumab. Some other parameters including the subsequent treatment proportions had a slight impact on the model outcomes.

**FIGURE 3:**
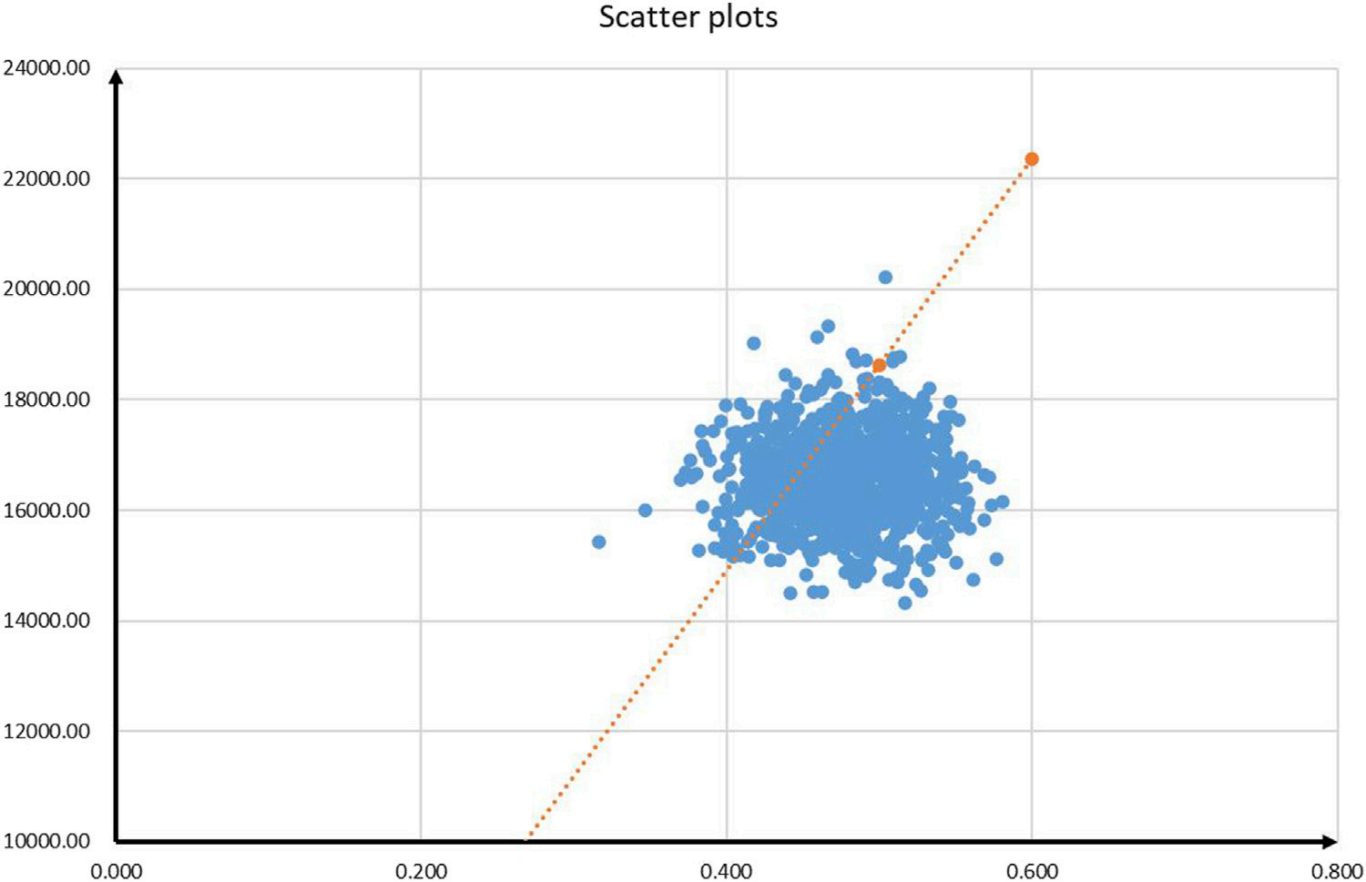
Cost-effectiveness scatter plots.

**FIGURE 4:**
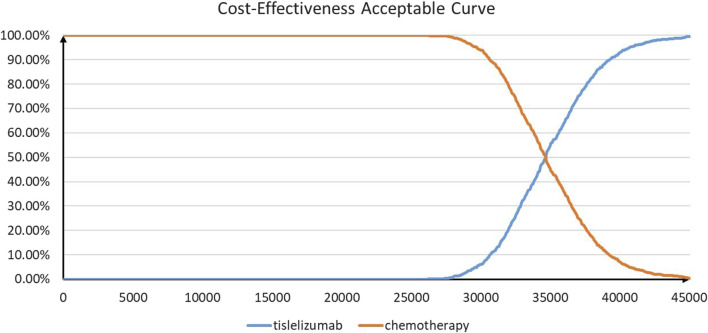
Cost-effectiveness acceptable curve.

The results of PSA are shown in Figures 3 and 4. According to the cost-effectiveness scatter plot (Figure 3), when WTP was set as $37,260 per QALY, it was obvious that most ICER points were placed under the line of WTP. In addition, the combination therapy had a probability of 77% to be cost-effective. According to the cost-effectiveness acceptability curve (Figure 4), we found that when the WTP threshold was set lower than $34,650 per QALY, tislelizumab plus chemotherapy were unlikely to be cost-effective. When the WTP threshold surpassed $30,300 per QALY, tislelizumab plus chemotherapy had a >50% probability to be cost-effective.

## Discussion

OSCC is a common malignant tumor all over the world. For patients diagnosed with advanced or metastatic OSCC, radiotherapy and chemotherapy can offer limited survival benefits. In recent days, ICIs have significantly altered this situation, which can offer patients significantly prolonged survival time. In China, several PD-1/PD-L1 inhibitors have been approved to treat patients with advanced or metastatic OSCC, and some of them are already included in the National Reimbursement Drug List (NRDL) (Liu et al., 2023). Several published economic evaluations have assessed the cost-effectiveness of camrelizumab, nivolumab, pembrolizumab, and sintilimab compared to chemotherapy in the first-line setting for Chinese patients with advanced or metastatic OSCC (Zhang et al., 2021; Liu et al., 2022; Shao et al., 2022; Ye et al., 2022; Zhu et al., 2022; Ye et al., 2023). One recently published study comprehensively evaluates the cost-effectiveness of currently available first-line immunotherapies for patients with advanced or metastatic OSCC from the perspective of the Chinese healthcare system (Liu et al., 2023). However, no current published studies have focused on tislelizumab. Therefore, our results could provide evidence for physicians to consider the cost-effectiveness of tislelizumab for advanced or metastatic OSCC. Decision-makers can also be informed whether the tislelizumab is cost-effective to be brought into the market.

This study is the first study to compare the cost-effectiveness of tislelizumab plus chemotherapy with placebo plus chemotherapy in patients with advanced or metastatic OSCC. Our base-case analysis revealed that tislelizumab combination therapy could bring additional QALYs despite there being additional costs. When the WTP threshold was set as $37,260 per QALY, the tislelizumab combination therapy was more cost-effective than chemotherapy with a probability of 77%. The subgroup population with a TAP score ≥10% had similar conclusions, while people with a TAP score <10% could not obtain cost-effectiveness from using the novel therapy. Based our DSA results, the base-case analysis results were sensitive to utilities of PFS and PD, which may lead to the change of conclusions. Other parameters like the cost of salvage therapy, laboratory tests and radiological examinations, and tislelizumab, as well as the subsequent therapy proportions had no significant impacts on the results.

According to DSA, the model outcomes were sensitive to the utilities of PFS and PD, which was similar to the findings of some published articles (Shao et al., 2022; Xu K. et al., 2023; Fang et al., 2023). We extracted the values of utilities from a published economic evaluation study that targeted the cost-effectiveness of the continuation *versus* discontinuation of first-line chemotherapy in patients with metastatic OSCC (Marguet et al., 2021). This might lead to some biases because the patients in the original study were not from China. Our DSA results revealed that if these two values fluctuate, ICERs might exceed the given WTP threshold. This problem could be solved after the health-related outcomes of RATIONALE-306 are reported. In addition, patients with different levels of TAP scores were key subgroups in RATIONALE-306. According to our subgroup analysis results, we found that patients in both the subgroups could gain significantly improved health benefits. However, only patients with a TAP score ≥10% could achieve cost-effectiveness. Thus, for advanced or metastatic OSCC patients with higher expression of PD-L1, tislelizumab plus chemotherapy can be recommended.

There are several limitations to the present study that should be emphasized. First, since there are no head-to-head studies, we use a placebo as the comparator instead of considering other first-line ICIs (e.g., camrelizumab). Future studies should focus on comparing the effectiveness of tislelizumab and other ICIs.

Second, due to lack of data, a budget-impact analysis (BIA) could not be conducted in this study. A BIA that can contextualize the potential total system affordability impact for policymakers should be conducted in the future to improve this study. Third, this cost-effectiveness analysis was conducted based on the interim analysis of RATIONALE-306. Therefore, some biases might exist in the survival extrapolation outcomes and adverse events. Future final analysis results of RATIONALE-306 can help to improve this study. Fourth, only costs and disutilities of grade ≥3 AEs were considered in this study. However, DSA results could confirm that this limitation may not have significant impacts on the results. Fifth, we extracted the utilities of PFS and PD from other trials. Our results were sensitive to these two values. Future health-related outcomes of RATIONALE-306 are needed to improve this study.

## Conclusion

Tislelizumab plus chemotherapy is likely to be a cost-effective option for patients with advanced or metastatic OSCC from the perspective of the Chinese healthcare system. In particular, patients with high expression of PD-L1 could achieve favorable cost-effectiveness from this novel combination therapy. However, long-term follow-up data are needed to improve this study.

## Data Availability

The original contributions presented in the study are included in the article/Supplementary Material; further inquiries can be directed to the corresponding author.
