# Supplementary material for: Cost-effectiveness analysis of tislelizumab plus chemotherapy as the first-line treatment for advanced or metastatic esophageal squamous cell carcinoma in China
Source: Front Pharmacol. 2024 May 15;15:1225076. doi: 10.3389/fphar.2024.1225076 (PMC11135043; doi:10.3389/fphar.2024.1225076)
Supplement: Supplementary file 1 [file DataSheet1.docx]

**Cost-Effectiveness Analysis of Tislelizumab Plus Chemotherapy as First-Line Treatment for Chinese Advanced or Metastatic Esophageal Squamous Cell Carcinoma**

Yanhong Liu^1^, Rong Shao^1#^

^1^ School of International Pharmaceutical Business, China Pharmaceutical University, Nanjing, China

#Correspondence:

Rong Shao

E-mail: shaorong118@163.com

**Content**

[Supplementary Table 1 2](#_Toc9168)

[Supplementary Table 2 3](#_Toc12957)

[Supplementary figure 1 4](#_Toc15064)

[Supplementary figure 2 5](#_Toc30519)

[Supplementary figure 3 6](#_Toc10778)

[Supplementary figure 4 7](#_Toc25569)

### Supplementary Table 1

| Tis_OS | | | | |  | Tis_PFS | | | | |
| --- | --- | --- | --- | --- | --- | --- | --- | --- | --- | --- |
| Model | LnL | Params | AIC | Rank |  | Model | LnL | Params | AIC | Rank |
| exponential | -103.82 | 1.00 | 209.65 | 7 |  | exponential | -125.05 | 1.00 | 252.10 | 7 |
| weibull | -96.04 | 2.00 | 196.08 | 4 |  | weibull | -123.24 | 2.00 | 250.48 | 6 |
| gamma | -95.05 | 2.00 | 194.11 | 2 |  | gamma | -119.77 | 2.00 | 243.53 | 4 |
| log normal | -96.50 | 2.00 | 196.99 | 5 |  | log normal | -101.43 | 2.00 | 206.87 | 2 |
| gompertz | -100.48 | 2.00 | 204.96 | 6 |  | gompertz | -122.34 | 2.00 | 248.68 | 5 |
| **log logistic** | **-94.27** | **2.00** | **192.55** | 1 |  | **log logistic** | **-98.92** | **2.00** | **201.83** | 1 |
| gengamma | -94.32 | 3.00 | 194.65 | 3 |  | gengamma | -100.70 | 3.00 | 207.39 | 3 |
| Che_OS | | | | |  | Che_PFS | | | | |
| Model | LnL | Params | AIC | Rank |  | Model | LnL | Params | AIC | Rank |
| exponential | -118.14 | 1.00 | 238.27 | 6 |  | exponential | -119.95 | 1.00 | 241.89 | 6 |
| weibull | -114.07 | 2.00 | 232.15 | 5 |  | weibull | -112.11 | 2.00 | 228.23 | 5 |
| gamma | -111.10 | 2.00 | 226.20 | 4 |  | gamma | -106.59 | 2.00 | 217.18 | 4 |
| **log normal** | **-99.84** | **2.00** | **203.69** | 1 |  | **log normal** | **-93.14** | **2.00** | **190.29** | 1 |
| gompertz | -118.13 | 2.00 | 240.27 | 7 |  | gompertz | -119.85 | 2.00 | 243.69 | 7 |
| log logistic | -100.68 | 2.00 | 205.37 | 3 |  | log logistic | -94.04 | 2.00 | 192.09 | 2 |
| gengamma | -99.62 | 3.00 | 205.25 | 2 |  | gengamma | -93.42 | 3.00 | 192.83 | 3 |

### Supplementary Table 2

| PD_L1 TAP>=10% | | | | |  | PD_L1 TAP<10% | | | | |
| --- | --- | --- | --- | --- | --- | --- | --- | --- | --- | --- |
| Tis_OS | | | | |  | Tis_OS | | | | |
| Model | LnL | Params | AIC | Rank |  | Model | LnL | Params | AIC | Rank |
| exponential | -67.90 | 1.00 | 137.80 | 7 |  | exponential | -71.64 | 1.00 | 145.29 | 6 |
| weibull | -64.68 | 2.00 | 133.35 | 3 |  | weibull | -68.58 | 2.00 | 141.17 | 3 |
| gamma | -64.47 | 2.00 | 132.94 | 2 |  | **gamma** | **-68.36** | **2.00** | **140.71** | **1** |
| log normal | -66.52 | 2.00 | 137.03 | 6 |  | log normal | -70.77 | 2.00 | 145.55 | 7 |
| gompertz | -66.30 | 2.00 | 136.60 | 5 |  | gompertz | -70.04 | 2.00 | 144.07 | 5 |
| **log logistic** | **-64.27** | **2.00** | **132.55** | **1** |  | log logistic | -68.45 | 2.00 | 140.91 | 2 |
| gengamma | -64.47 | 3.00 | 134.94 | 4 |  | gengamma | -68.31 | 3.00 | 142.63 | 4 |
| Che_OS | | | | |  | Che_OS | | | | |
| Model | LnL | Params | AIC | Rank |  | Model | LnL | Params | AIC | Rank |
| exponential | -67.58 | 1.00 | 137.16 | 6 |  | exponential | -88.10 | 1.00 | 178.20 | 6 |
| weibull | -66.05 | 2.00 | 136.10 | 5 |  | weibull | -85.49 | 2.00 | 174.98 | 5 |
| gamma | -64.65 | 2.00 | 133.29 | 4 |  | gamma | -83.64 | 2.00 | 171.28 | 4 |
| log normal | -58.75 | 2.00 | 121.49 | 2 |  | **log normal** | **-77.00** | **2.00** | **157.99** | **1** |
| gompertz | -67.51 | 2.00 | 139.03 | 7 |  | gompertz | -88.10 | 2.00 | 180.20 | 7 |
| log logistic | -59.75 | 2.00 | 123.49 | 3 |  | log logistic | -77.70 | 2.00 | 159.41 | 3 |
| **gengamma** | **-57.31** | **3.00** | **120.62** | **1** |  | gengamma | -76.60 | 3.00 | 159.20 | 2 |

### Supplementary figure 1

Overall survival

| 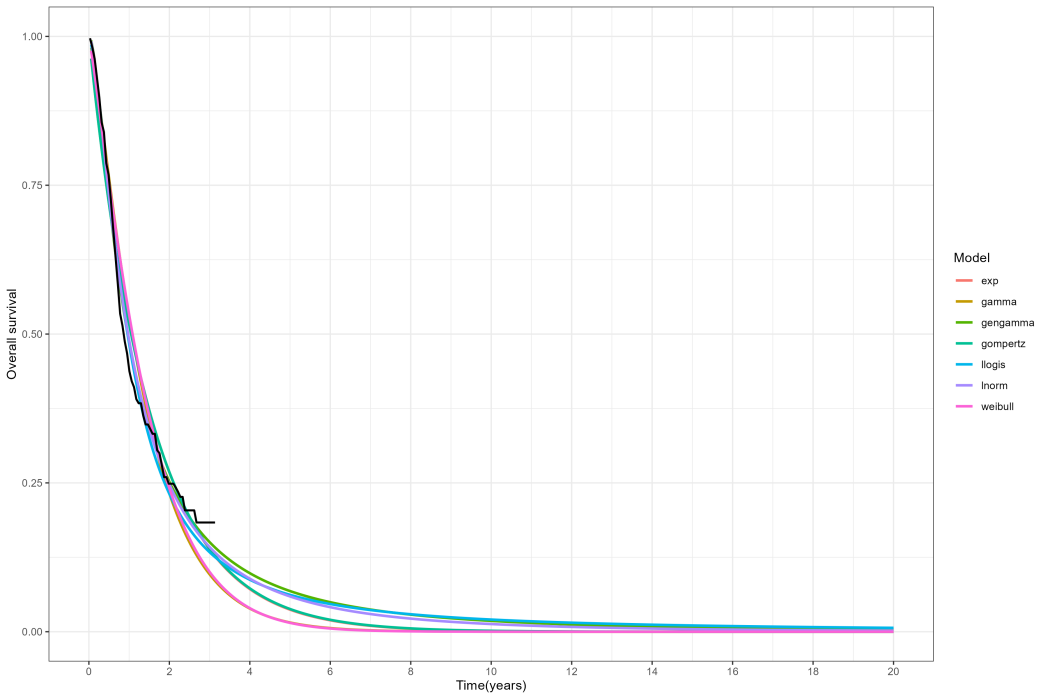 | 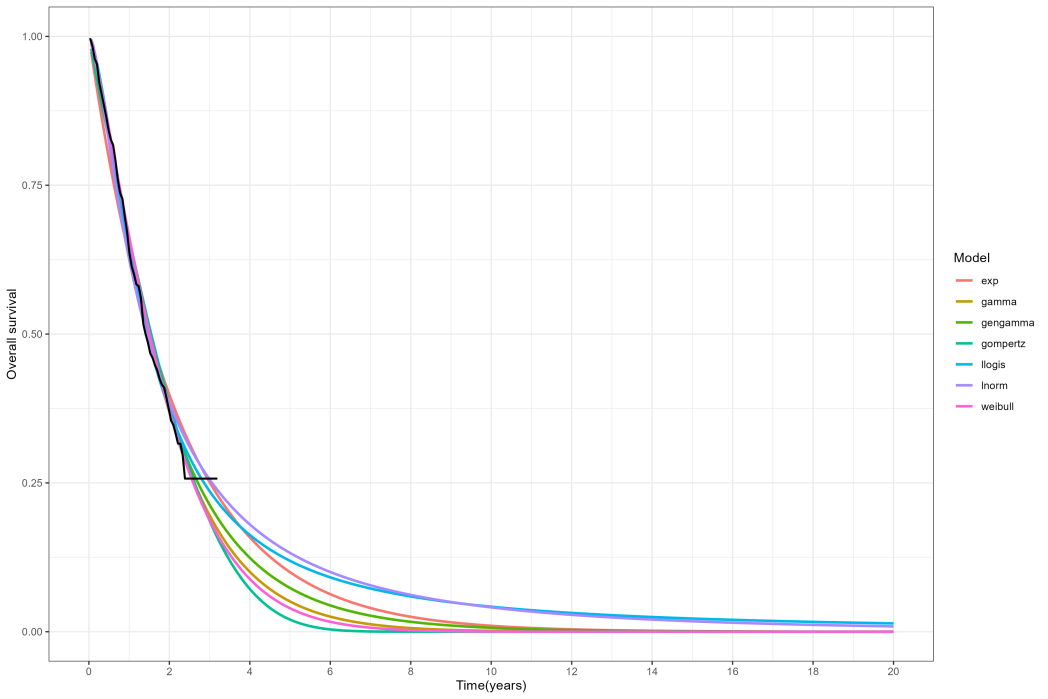 |
| --- | --- |
| Chemotherapy | Tislelizumab |

### Supplementary figure 2

Progression-free survival

| 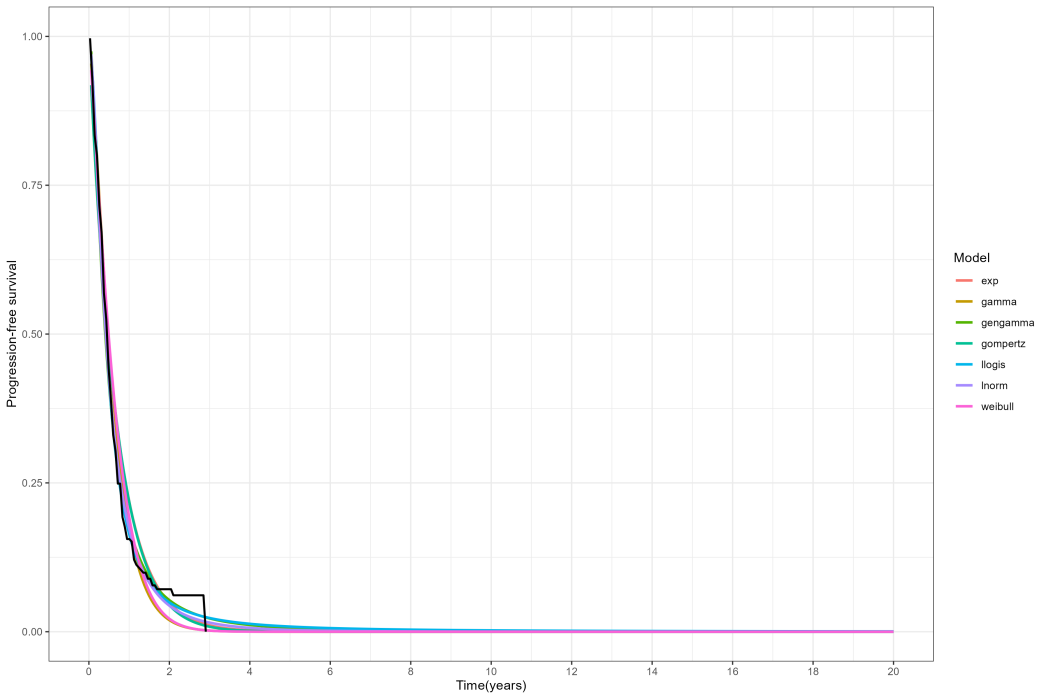 | 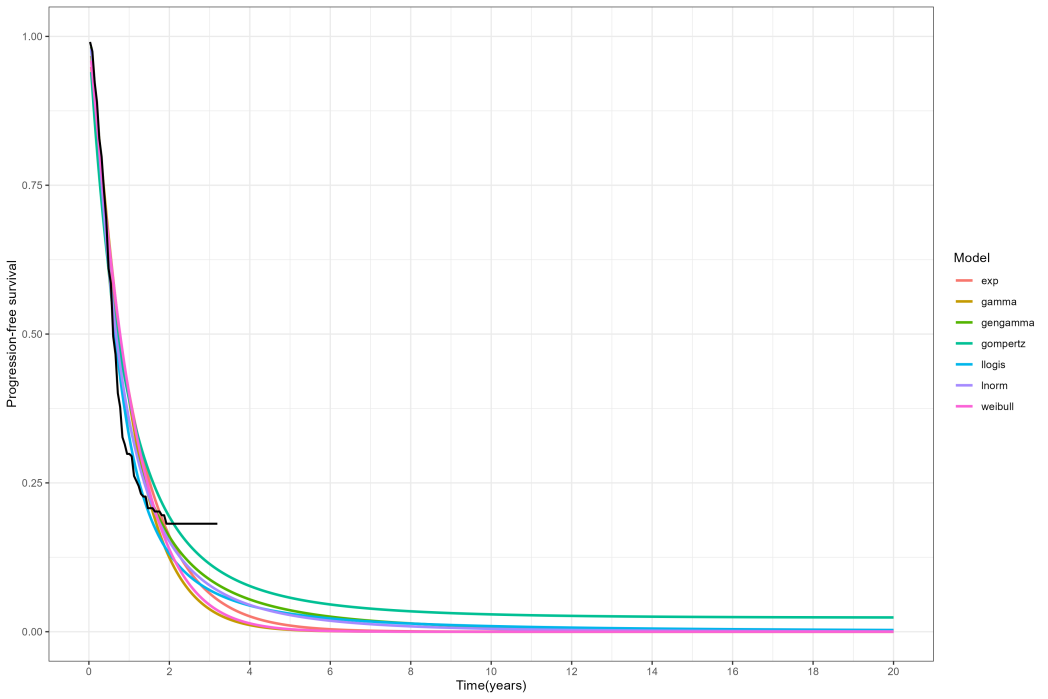 |
| --- | --- |
| Chemotherapy | Tislelizumab |

### Supplementary figure 3

Overall survival, PD_L1 TAP>=10%

| 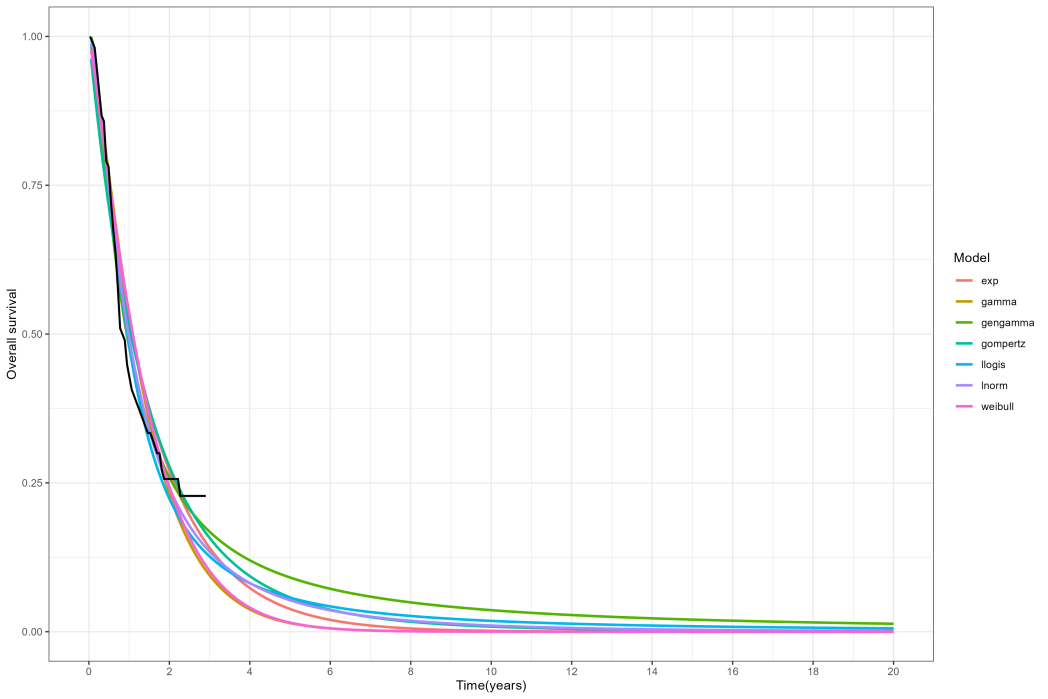 | 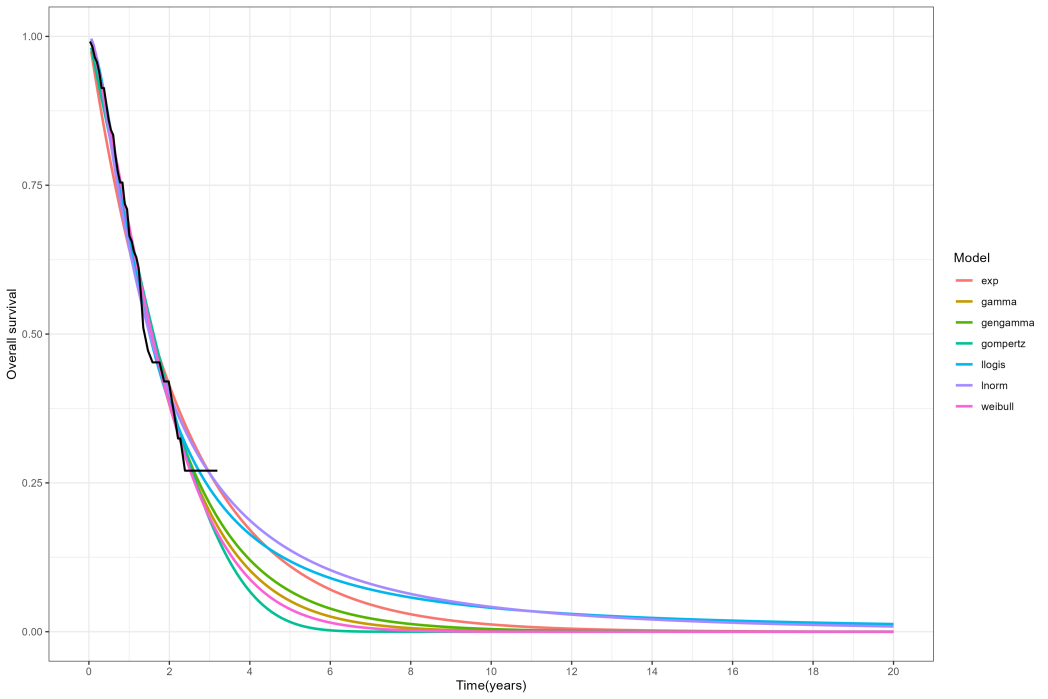 |
| --- | --- |
| Chemotherapy | Tislelizumab |

### Supplementary figure 4

Overall survival, PD_L1 TAP<10%

| 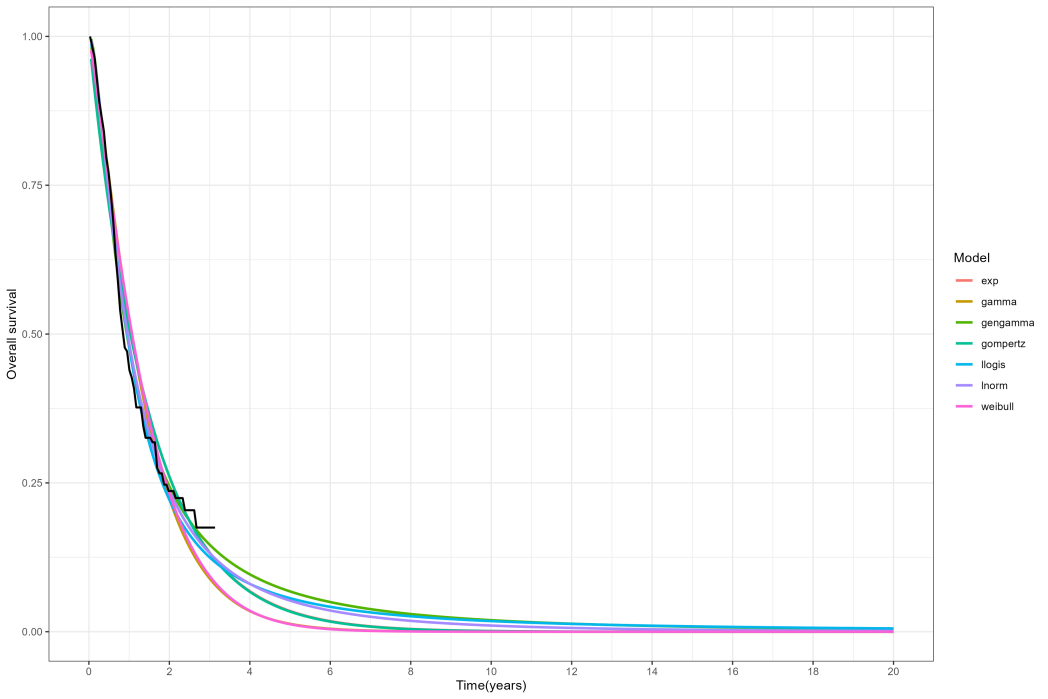 | 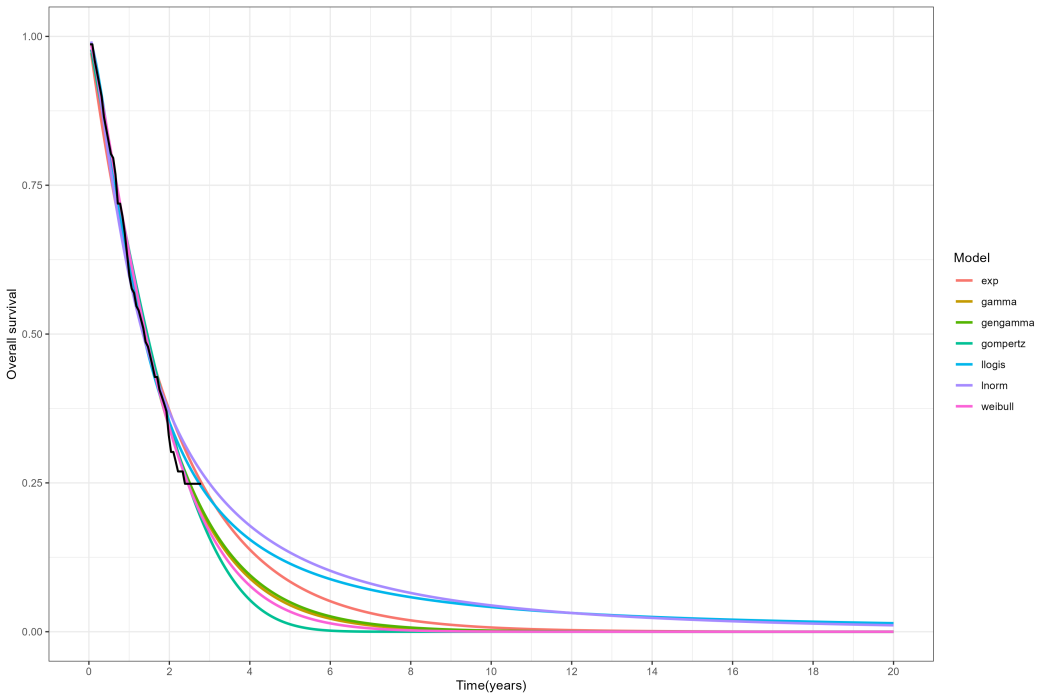 |
| --- | --- |
| Chemotherapy | Tislelizumab |
